# Miocardite Aguda após Vacina de mRNA contra a COVID-19: Uma Correspondência

**DOI:** 10.36660/abc.20220331

**Published:** 2022-11-23

**Affiliations:** 1 Private Academic Consultant Bangkok Tailândia Pesquisador autônomo, Bangkok – Tailândia; 2 Dr. DY Patil University Pune Índia Dr. DY Patil University, Pune – Índia

**Keywords:** Miocardite, mRNA, COVID-19, Vacina

**Caro editor,**

Gostaríamos de compartilhar ideias sobre a publicação “Miocardite aguda após vacina de mRNA contra a COVID-19”.^1^ Gomes et al. examinan os potenciais efeitos colaterais da vacinação COVID-19 e o estado atual da miocardite aguda. A imunização para COVID-19 provavelmente causará anormalidades cardíacas. Concordamos que esa é uma possibilidade. No entanto, deve-se notar que muitas vezes há informações inadequadas sobre o estado de saúde/cardíaco dos casos problemáticos antes da imunização. Embora uma análise pós-vacinação, que inclua um eletrocardiograma, possa confirmar a presença de miocardite, é impossível estabelecer uma ligação entre a doença cardíaca e a vacina para COVID-19. Também existe a possibilidade de você ter problemas médicos simultaneamente. A dengue, por exemplo, pode induzir problemas cardíacos se ocorrer concomitantemente.^2^

## References

[1] Gomes DA, Santos RR, Freitas P, Paiva MS, Ferreira J, Trabulo M (2022). Acute Myocarditis Following mRNA COVID-19 Vaccine. Arq Bras Cardiol.

[2] Kebayoon A, Wiwanitkit V (2021). Dengue after COVID-19 Vaccination: Possible and Might be Missed. Clin Appl Thromb Hemost.

