# Supplementary material for: Miocardite Aguda após Vacina de mRNA contra a COVID-19: Uma Correspondência
Source: Arq Bras Cardiol. 2022 Nov 23;119(6):1006–7. [Article in Portuguese] doi: 10.36660/abc.20220331 (PMC9814794; doi:10.36660/abc.20220331)
Supplement: Translation

# Translation: Acute Myocarditis after mRNA COVID-19 Vaccine: A Correspondence

**Affiliations:** 1 Bangkok Thailand Autonomous Researcher, Bangkok – Thailand; 2 Pune India Dr. DY Patil University, Pune – India

**Keywords:** Myocarditis, mRNA, COVID-19, Vaccine

**Dear Editor,**

We would like to share ideas on the publication “Acute Myocarditis Following mRNA COVID-19 Vaccine.”^1^ Gomes et al. examine the potential side effects of COVID-19 vaccination and the current state of acute myocarditis. COVID-19 immunization will probably cause heart abnormalities. We agree that this is a possibility. However, it should be noted that there is frequently inadequate information on the health/cardiac status of the troublesome cases before immunization. Although a post-vaccination analysis, which includes an EKG, can confirm the presence of myocarditis, it is impossible to establish a link between heart disease and the COVID-19 vaccine. There is still a potential that you will have medical issues simultaneously. Dengue fever, for example, can induce cardiac problems if it co-occurs.^2^
