# Supplementary material for: Miocardite Aguda após Vacina de mRNA contra a COVID-19: Uma Correspondência
Source: Arq Bras Cardiol. 2022 Nov 23;119(6):1006–7. [Article in Portuguese] doi: 10.36660/abc.20220331 (PMC9814794; doi:10.36660/abc.20220331)
Supplement: Related texts

# Reply: Carta-resposta

**Authors:** Daniel A. Gomes, Rita R. Santos, Pedro Freitas, Mariana S. Paiva, Jorge Ferreira, Marisa Trabulo

**Affiliations:** 1 Departamento de Cardiologia Hospital de Santa Cruz Centro Hospitalar de Lisboa Ocidental Carnaxide Portugal Departamento de Cardiologia, Hospital de Santa Cruz, Centro Hospitalar de Lisboa Ocidental, Carnaxide – Portugal

**Caro editor,**

Agradecemos a Mungmunpuntipantip e Wiwanitkit por sua carta sobre nosso artigo intitulado “ Miocardite aguda após vacina de mRNA contra a COVID-19”.^1^

Concordamos que embora haja uma relação temporal entre a vacinação e o desenvolvimento de miocardite, não é possível estabelecer causalidade. No entanto, após relatarmos este caso clínico, foram publicadas séries de casos de miocardite após a vacina BNT162b2b mRNA (Pfizer-BioNTech).^2,3^

No relato de Mevorach et al.,^2^ com 136 casos de miocardite pós-vacinação, o risco observado foi 2,35 vezes maior em relação a pessoas não vacinadas, e ocorreu um caso fatal. Em outro relato com 54 casos de Witberg et al.,^3^ a incidência de miocardite até 42 dias após a vacinação foi de 2,13 por 100.000 pessoas, todas com recuperação espontânea. Em ambas as séries, a maior incidência foi entre os jovens do sexo masculino.

Ressalta-se que a ocorrência de miocardite após a vacinação para COVID-19 é rara e autolimitada na grande maioria dos casos.^4^ Portanto, esta suposta complicação não altera a relação benefício/risco superior da vacina.

## References

[1] Gomes DA, Santos RR, Freitas P, Paiva MS, Ferreira J, Trabulo M (2022). Acute Myocarditis Following mRNA COVID-19 Vaccine. Arq Bras Cardiol.

[2] Mevorach D, Anis E, Cedar N, Bromberg M, Haas EJ, Nadir E (2021). Myocarditis after BNT162b2 mRNA Vaccine Against Covid-19 in Israel. N Engl J Med.

[3] Witberg G, Barda N, Hoss S, Richter I, Wiessman M, Aviv Y (2021). Myocarditis after Covid-19 Vaccination in a Large Health Care Organization. N Engl J Med.

[4] Caforio ALP (2021). Receipt of mRNA Vaccine against Covid-19 and Myocarditis. N Engl J Med.

# Reply

**Authors:** Daniel A. Gomes, Rita R. Santos, Pedro Freitas, Mariana S. Paiva, Jorge Ferreira, Marisa Trabulo

**Affiliations:** 1 Departamento de Cardiologia Hospital de Santa Cruz Centro Hospitalar de Lisboa Ocidental Carnaxide Portugal Departamento de Cardiologia, Hospital de Santa Cruz, Centro Hospitalar de Lisboa Ocidental, Carnaxide – Portugal

**Dear Editor,**

We thank Mungmunpuntipantip and Wiwanitkit for their letter regarding our paper entitled “Acute Myocarditis Following mRNA COVID-19 Vaccine”.^1^

We agree that although there is a temporal relationship between vaccination and the development of myocarditis, it is not possible to establish causality. However, after we reported this clinical case, case series were published regarding myocarditis following the BNT162b2b mRNA vaccine (Pfizer-BioNTech).^2,3^

In the report by Mevorach et al.,^2^ with 136 cases of post-vaccination myocarditis, the observed risk was 2.35 times higher compared to unvaccinated persons, and one fatal case occurred. In another report with 54 cases by Witberg et al.,^3^ the incidence of myocarditis up to 42 days following vaccination was 2.13 per 100.000 persons, all experiencing spontaneous recovery. In both series, the highest incidence was amongst young males.

It should be outlined that the occurrence of myocarditis following COVID-19 vaccination is rare and self-limited in the vast majority of cases.^4^ Therefore, this presumed complication must not alter the vaccine's superior benefit/ risk relationship.
